# Characterizing the immune microenvironment of malignant peripheral nerve sheath tumor by PD-L1 expression and presence of CD8+ tumor infiltrating lymphocytes

**DOI:** 10.18632/oncotarget.11734

**Published:** 2016-08-31

**Authors:** Elizabeth Shurell, Arun S. Singh, Joseph G. Crompton, Sarah Jensen, Yunfeng Li, Sarah Dry, Scott Nelson, Bartosz Chmielowski, Nicholas Bernthal, Noah Federman, Paul Tumeh, Fritz C. Eilber

**Affiliations:** ^1^ Division of Surgical Oncology, Department of Surgery, University of California, Los Angeles, CA 90095, USA; ^2^ Department of Hematology/Oncology, University of California, Los Angeles, CA 90095, USA; ^3^ Department of Pathology and Laboratory Medicine, University of California, Los Angeles, CA 90095, USA; ^4^ Department of Orthopaedic Surgery, University of California, Los Angeles, CA 90095, USA; ^5^ Department of Dermatology, University of California, Los Angeles, CA 90095, USA; ^6^ Department of Molecular and Medical Pharmacology, University of California, Los Angeles, CA 90095, USA; ^7^ UCLA JCCC Sarcoma Program, University of California, Los Angeles, CA 90095, USA

**Keywords:** immune microenvironment, MPNST, PD-L1, CD8, sarcoma

## Abstract

**Background:**

Malignant peripheral nerve sheath tumor (MPNST) is an aggressive sarcoma with few treatment options. Tumor immune state has not been characterized in MPNST, and is important in determining response to immune checkpoint blockade. Our aim was to evaluate the expression of programmed death-ligand 1 (PD-L1), programmed cell death protein 1 (PD-1), and presence of CD8+ tumor infiltrating lymphocytes (TILs) in MPNST, and correlate these findings with clinical behavior and outcome.

**Results:**

PD-L1 staining of at least 1% was seen in 0/20 nerves, 2/68 benign lesions and 9/53 MPNST. Two of 68 benign lesions and 7/53 (13%) MPNST had at least 5% PD-L1 staining. CD8 staining of at least 5% was seen in 1/20 (5%) nerves, 45/68 (66%) benign lesions and 30/53 (57%) MPNST. PD-L1 was statistically more prevalent in MPNST than both nerves and benign lesions (p=0.049 and p=0.008, respectively). Expression of PD-1 was absent in all tissue specimens. There was no correlation of PD-L1 or CD8 expression with disease state (primary versus metastatic) or patient survival.

**Methods:**

A comprehensive PNST tissue microarray was created from 141 surgical specimens including primary, recurrent, and metastatic MPNST (n=53), neurofibromas (n=57), schwannoma (n=11), and normal nerve (n=20). Cores were stained in triplicate for PD-L1, PD-1, and CD8, and expression compared between tumor types. These data were then examined for survival correlates in 35 patients with primary MPNST.

**Conclusions:**

MPNST is characterized by low PD-L1 and absent PD-1 expression with significant CD8+ TIL presence. MPNST immune microenvironment does not correlate with patient outcome.

## INTRODUCTION

Malignant peripheral nerve sheath tumor (MPNST) is a soft tissue sarcoma that arises from neurofibromas, particularly plexiform neurofibromas, or in association with a large nerve without an identifiable neurofibroma component. MPNST arise spontaneously, or can occur in association with neurofibromatosis type 1 (NF-1), a disease characterized by the loss of the neurofibromin tumor suppressor protein. Patients with NF-1 have a 10% lifetime risk of developing MPNST. MPNST often present as an enlarging, painful mass, and are further characterized on imaging with either CT or MRI. In the case of NF1-associated MPNST, discerning malignancy from benign neurofibromas is a clinical challenge that has led to the utilization of PET imaging in tumor diagnosis [1].

MPNST are best managed by multidisciplinary teams at tertiary sarcoma referral centers [2]. Surgery is the foundation of therapy for MPNST, however a major unmet clinical need is in patients who are poor surgical candidates or have relapsed/systemic disease. MPNST remain chemo-resistant, and long term outcomes are poor. Recent developments in molecular biology have uncovered alterations in several important growth and developmental pathways in this disease including the Ras-MAPK pathway, the Wnt-Beta-Catenin pathway, the mTOR pathway, and in PTEN and p53 [3]. However, targeted therapies to date have not been very efficacious; as such new therapeutic modalities are needed.

Immune-based therapies have shown significant therapeutic efficacy in numerous tumor types, including advanced melanomas and non-small cell lung cancers.[4–7] Immune-induced tumor programmed cell death-ligand 1 (PD-L1) expression is considered to be an adaptive immune resistance mechanism for tumor cells in response to immune challenge.[8–10] Sufficient T cell infiltration is essential for response to PD-L1 blockade. The presence of tumor escape or suppression of tumoral immune response has not been well studied in patients with sarcoma. In this study, we characterized the expression of programmed cell death protein 1 (PD-1), PDL-1 and CD8 tumor infiltrates in MPNST, neurofibromas, schwannomas, and in normal nerve tissue, and correlated these factors with patient outcome.

## RESULTS

### Patient characteristics

Of the patients with MPNST (n=53), 33 (62.3%) were spontaneous tumors and 20 (37.7%) were NF-1 associated MPNST. Most patients had primary (71.7%, n=38), high grade (83.0%, n=44), large tumors (mean 11.9 cm, median 10 cm, range 1-45cm) located on the extremity (43.4%, n=23). (Table 1) The majority of MPNST patients in this study were not treated with neoadjuvant chemotherapy (58.5%, n=31) or neoadjuvant radiation therapy (69.8%, n=37) before the surgical specimen was retrieved. Median follow-up time for MPNST survivors was 6.1 years (range 2.3-15.8 years). The primary site of metastatic disease, when it occurred in this MPNST patient cohort, was the lung.

**Table 1:** Patient Characteristics

MPNST Patient Characteristics			
		n	%
**Total MPNST patients**		53	100
**Genetic Background**			
	Spontaneous	33	62.3
	NF-1 associated	20	37.7
**Tumor Type**			
	Primary	38	71.7
	Recurrent	10	18.9
	Metastatic	5	9.4
**Grade**			
	High	44	83.0
	Intermediate	8	15.1
	Low	1	1.9
**Location**			
	Extremity	23	43.4
	Pelvic/Retroperitoneal	16	30.2
	Trunk	7	13.2
	Head and Neck	5	9.4
	Other	2	3.8
**Neoadjuvant Chemotherapy**			
	Yes	22	41.5
	No	31	58.5
**Neoadjuvant Radiation**			
	Yes	16	30.2
	No	37	69.8
**Tumor Size**			
	Mean	11.9cm	
	Median	10cm	
	Range	1-45cm	
**Follow up**			
	Mean	6.1 years	
	Range	2.3-15.8 years	

### PD-L1 expression is significantly higher in MPNST when compared to benign tumors

**Figure 1:**
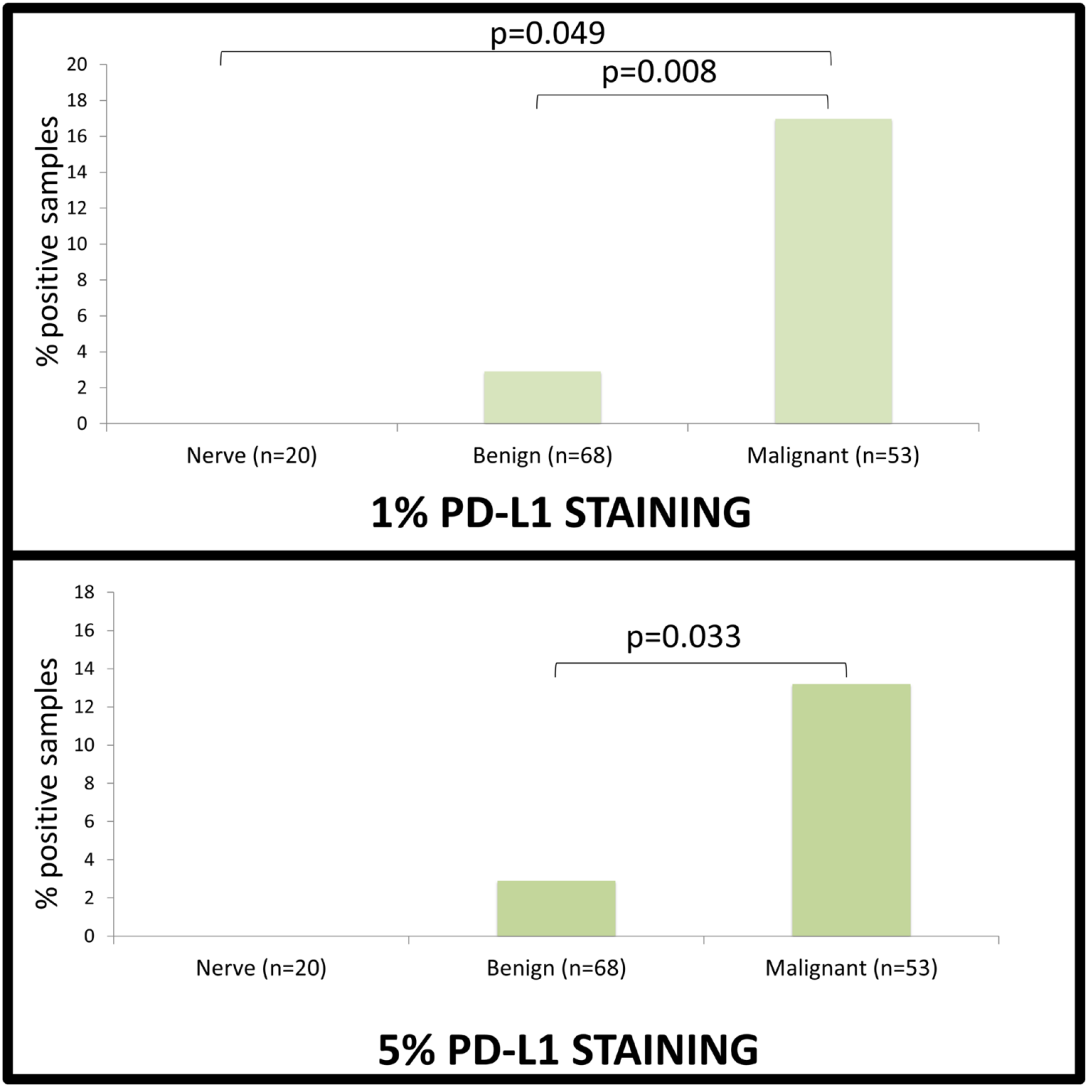
PD-L1 Expression 1-5 cores from different blocks were stained for PD-L1, PD-1 and CD8^+^ and scored based on intensity of staining on a scale of 0-3 and percent of cells staining positively. Significance of staining differences between groups was compared via chi squared analysis and survival analysis was performed using a Cox proportional hazards model. PD-L1 staining of at least 1% was seen in 0/20 nerves, 2/68 benign lesions and 9/53 malignant lesions. PD-L1 staining of at least 5% was seen in 0/20 nerves, 2/68 benign lesions and 7/53 malignant lesions. PD-L1 was statistically more prevalent in MPNST than both nerves and benign lesions (p=0.049 and p=0.008, respectively) at the 1% level, but only benign lesions at the 5% level (p=0.033).

**Figure 2:**
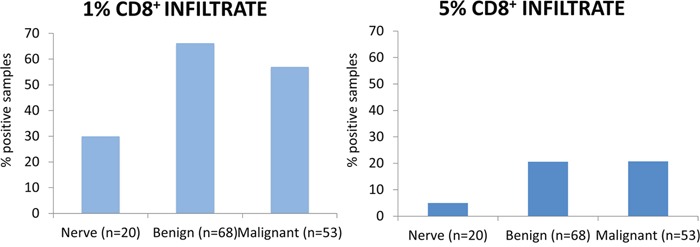
CD8^+^ infiltrate CD8^+^ of at least 1% was seen in 6/20 (30.0%) nerves, 45/68 (66.2%) benign lesions and 30/53 (56.6%) of MPNST. CD8^+^ of at least 5% was seen in 1/20 (5.0%) nerves, 14/68 (20.6%) benign lesions and 11/53 (20.7%) of MPNST. There was no difference between benign and malignant tumors at the 5% (p=0.282) or 1% (p=0.982) expression threshold.

**Figure 3:**
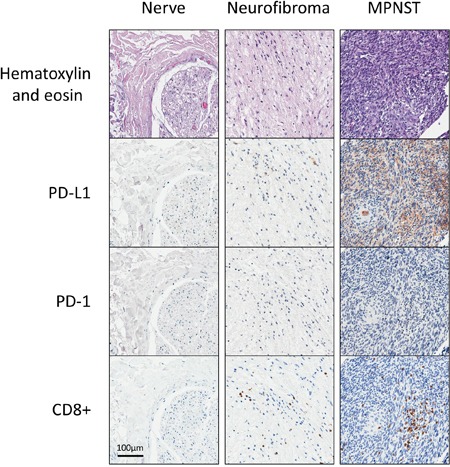
Representative immunohistochemical stainings of PD-L1, PD-1, and CD8^+^ for nerve, benign peripheral nerve sheath tumors, and malignant peripheral nerve sheath tumors

To examine the immune microenvironment of benign and malignant tissues, we quantified PD-L1 expression and CD8 positivity in a wide array of patient samples using 1% and 5% positive threshold criteria. We found no difference in expression of PD-L1 in normal nerve as compared to benign neurofibromas and schwannomas (p=0.438). CD8+ immune infiltrates, however, were significantly elevated in benign peripheral nerve sheath tumor (45/68 benign PNST) as compared to normal nerve (6/20 nerves) using a 1% threshold, p=0.004. In the comparison of normal nerves to MPNST, malignant tumor samples exhibited significantly higher PD-L1 expression (9/53 MPNST samples versus 0/20 nerve samples; p=0.049, Figure 1). CD8 immune infiltrates were present in a significantly higher proportion in MPNST tumors (30 of 53 samples) as compared to normal nerve (6 of 20 samples, p=0.043, see Figure 2). PD-1 showed no staining in any tissue specimens (Figure 3).

Using a more stringent 5% threshold for positivity, MPNST tumors had a significantly higher prevalence of PD-L1 expression than benign peripheral nerve sheath tumors (2 of 68 benign PNST; 7 of 53 MPNST; p=0.033). There was no difference in the presence of CD8+ immune infiltrates between benign PNST and MPSNT using a 5% threshold (p=0.282, see Figure 2).

### Comparison of the tumor immune microenvironment in localized and metastatic MPNST

To characterize the tumor immune microenvironment as a MPNST progresses from primary to metastatic tumor, we examined PD-L1 expression and the presence of CD8 immune infiltrates in 38 primary MPNST samples, 10 recurrent MPNST samples, and 5 metastatic MPNST samples. There was no significant difference in PD-L1 expression between primary (18%), recurrent (20%), or metastatic (0%) samples (Table 2). When examining the presence of CD8 immune infiltrates, there was no significant difference between primary (53%), recurrent (80%), and metastatic samples (40%) (Table 2).

**Table 2:** Expression of CD8^+^ and PD-L1 in primary, recurrent, and metastatic MPNST

Primary MPNST	Recurrent MPNST		Metastatic MPNST	
**PD-L1**	PD-L1 (1%) = 2/10	PD-L1 (5%) = 2/10	PD-L1 (1%) = 0/5	PD-L1 (5%) = 0/5
PD-L1 (1%) = 7/38	p=0.909		p=0.294	
PD-L1 (5%) = 5/38		p=0.585		p=0.388
**CD8+**	CD8+ (1%) = 8/10	CD8+ (5%) = 2/10	CD8+ (1%) = 2/5	CD8+ (5%) = 0/5
CD8+ (1%) = 20/38	p=0.118		p=0.595	
CD8+ (5%) = 9/38		p=0.805		p=0.221

No significant differences were noted in the expression level of PD-L1 or the amount of CD8+ lymphocytes between primary and recurrent or metastatic MPNST samples.

### PD-L1 expression and CD8 TIL co-presence

Given that PD-L1 is inducible and may reflect homeostatic responses to immune activation [6], characterizing the immune infiltrate is important in contextualizing tumoral PD-L1 expression. Both CD4 and CD8 cell expression have prognostic implications in soft tissue sarcoma.[11, 12] Therefore, we examined the synchronous presence of PD-L1 expression and CD8 TILs in all neurogenic tissues. Of the samples with both strong PD-L1 and CD8 expression, 100% were in patients with malignant tumors. Of these nine patients, four were in primary MPNST samples, two were in MPNST recurrent tumors, two were from primary MPNST tumors in patients with concomitant metastases, and one was in a neurofibroma sample adjacent to a primary MPNST.

### Significance of PD-L1 expression and CD8 positivity on MPNST patient survival

We explored patient disease specific survival (DSS) and disease free survival (DFS) in relation to PD-L1 and CD8 levels. Thirty-eight patients with primary MPNST tumors were included in the tissue microarray. Three of the samples came from the primary tumor of patients who also had concurrent metastases, and were therefore excluded from the analysis. Median DSS was 24 months (range 3 months - 15 years). Of the 35 remaining patients with primary MPNST, DSS analysis revealed no survival correlates to PD-L1 or CD8 expression (PD-L1 5% criteria: NS, p=0.717; PD-L1 1% criteria: NS, p=0.342; CD8 5% criteria: NS, p=0.459; CD8 1% criteria: NS, p=0.938).

Analysis of DFS was limited to 33 patients, as two patients with primary non-metastatic MPNST had DFS less than one month from surgery and were censored from the analysis. Of the 33 patients with primary MPNST who underwent R0 surgical resection, DFS did not correlate with PD-L1 nor CD8 expression (PDL1 5% criteria: NS, p=1; PDL1 1% criteria: NS, p=0.630, CD8 5% criteria: NS, p=0.553; CD8 1% criteria: NS, p=0.109).

## DISCUSSION

The immune system has the capacity to recognize and eliminate cancer cells, but can be restrained by inhibitory mechanisms in the tumor environment. To date the immunosuppressive effects of the MPNST tumor microenvironment are still largely unknown [13]. Immune checkpoint pathways maintain self-tolerance and limit damage to host tissues during an immune response, but these pathways can be exploited by cancer cells to evade immune destruction [10]. Monoclonal antibodies interrupting immune checkpoints, such as anti-CTLA-4, anti-PD-1, and anti-PD-L1 can unleash anti-tumor immunity and mediate durable cancer regressions. While the presence of PD-L1 expression is considered the best available biomarker for PD-L1/PD-1 blockade, several other predictive biomarkers of response to checkpoint blockade are currently being explored.[14–17].

Programmed-death (PD) pathway blockade has resulted in significant and durable clinical responses in patients with a broad spectrum of so-called “inflamed cancers”—such as melanoma, renal cell carcinoma, lung cancer, mismatch repair-deficient colorectal cancer, and bladder cancer—characterized by a high prevalence of neo-antigens, elevated PD-L1 expression, and robust infiltration of cytotoxic T cells (see review of “inflamed cancer” by Zou et al.) [18]. On the other hand, “non-inflamed” cancers with poor T cell trafficking and low PD pathway activity are thought to be less susceptible to single-agent PD pathway immunotherapy and may require combinatorial regimens in order to induce a clinically-meaningful response. Characterizing the immunologic microenvironment of malignant peripheral nerve sheath tumors (MPNST) as “inflamed” or “non-inflamed” is therefore important because it may lend insight into whether patients with advanced MPNST respond to single-agent PD pathway blockade or may indicate when alternative immunotherapeutic approaches such as adoptive transfer of T cells or combination immunotherapy may be more appropriate.

The principal aim of this study was to characterize the immunologic microenvironment of MPNST by quantifying infiltration of cytotoxic CD8+ T cells and measuring the expression of programmed death-1 (PD1) and programmed-death-1 ligand-1 (PD-L1). Given that the immunologic microenvironment has prognostic significance in a variety of cancer histologies, we also sought to clarify whether cytotoxic T cell infiltration and PD1/PD-L1 expression correlated with disease progression and patient survival.

To evaluate PD pathway activity in MPNST, we used one of the largest prospective datasets of MPNST available which includes tumor specimens from 86 patients collected between 1982 and 2009 at a single institution. We found significant (57%) infiltration of cytotoxic T cells in the MPNST microenvironment but an absence of PD1 expression and low levels of PD-L1. The CD8+ T cell infiltration was essentially limited to MPNST with minimal infiltration in normal nerve and benign tumor (p=0.043). We also observed that only 9 out of 53 MPNST tumor specimens had expression of PD-L1, and only at relatively low levels (less than 1% of cells per high-power field). Furthermore, we found that disease-specific survival and disease-free survival among patients in our study were not associated with expression of PD-L1 or the presence of tumor-infiltrating CD8+ lymphocytes.

Previous studies evaluating the activity of the PD pathway in patients with MPNST and other sarcomas for that matter are limited by their small sample sizes and have produced mixed results. In a case series of 105 patients with various soft tissue sarcomas, Kim *et al.* found that 3 out of 6 patients with MPNST showed expression of both PD1 and PD-L1 in the tumor microenvironment [19]. In another case series of 50 patients with a variety of soft tissue sarcomas, one patient with MPNST was found to have an absence of PD-L1 expression on both tumor and lymphocytes [20].

It has been suggested that non-inflamed tumor types have prominent features of epithelial to mesenchymal transition and stem-like characteristics with a paucity of neo-antigens and multiple layers of immunosuppressive mechanisms. Of the 53 patients with MPNST in the present study, 44 (83%) had high-grade disease consistent with a non-inflamed phenotype. The emerging complexity of immunoregulatory mechanisms in non-inflamed cancers might limit the efficacy of single-agent PD pathway blockade and suggest that the most effective treatment for patients with MPNST may involve a combinatorial approach including enforcing T cell trafficking with epigenetic reprogramming drugs, supplementation of effector T cells with adoptive transfer, and subversion of other immunosuppressive elements such as T regulatory cells in the tumor microenvironment.

Current cytotoxic treatments of MPNST, such as adriamycin and ifosfamide, lack efficacy,[21] and the rarity of these tumors is a barrier to appropriately powered randomized controlled studies testing novel chemo- and immunotherapeutics [22]. To maximize the value of information derived from relatively small numbers of human tumors, the use of murine models may lend insight into MPNST pathogenesis and potential treatments. Murine models of MPNST are of important use for basic and translational research as they can mimic the clinical pattern of growth and metastasis.[23–25] Cross-species comparative oncogenomics may help to identify functionally validated molecular drivers for study in human MPNST, and patient-derived orthotopic xenografts may provide a platform for future testing of novel therapeutics.[26, 27] Thus, a potential area of further study is the examination of targeted chemotherapy and combinatorial PD pathway blockade in transgenic and patient-derived orthotopic xenograft MPNST models.

In conclusion, we assembled a large cohort of patients with MPNST to provide a broad view of the immunologic landscape of primary and metastatic tumors. The finding that MPNST tumors resemble “non-inflamed” cancers in terms of low PD activity and T cell infiltration has major therapeutic implications for how PD blockade may be supplemented with other immunotherapy modalities to develop a combinatorial approach to promote durable and potent anti-tumor immunity.

## MATERIALS AND METHODS

### Patients

The University of California – Los Angeles (UCLA) Comprehensive Cancer Center is one of the highest volume sarcoma programs in the nation. The Sarcoma Program at UCLA provides innovative multidisciplinary treatment for patients with sarcoma in all stages of disease. Since 1974, Sarcoma Program at the University of California – Los Angeles (UCLA) Comprehensive Cancer Center has prospectively maintained a peripheral nerve sheath tumor database with clinical and pathologic patient data. A protocol detailing the study design and analysis was approved by the UCLA Institutional Review Board (IRB) and the Jonsson Comprehensive Cancer Center. For inclusion, subjects were required to have tissue diagnosis of a peripheral nerve sheath tumor, undergone surgery and treatment of the tumor at UCLA, and have documented follow-up after surgery. 267 unique patients were eligible for study, of those 86 patients comprising 141 surgical specimens had tissue available for inclusion in the tissue microarray: 53 MPNST specimens, 57 neurofibromas, 11 schwannomas, and 20 normal nerve samples from a period of 27 years (1982-2009). When available, specimens of normal nerve and neurofibroma were sampled from MPNST resection specimens; additionally if a patient later underwent surgical resection of recurrent and/or metastatic MPNST, these tumors were also included in the microarray. Original surgical specimens were reviewed by a UCLA sarcoma pathologist (S.M.D.) to confirm pathologic diagnosis and grading. All medical records were reviewed to confirm accuracy of the prospectively maintained database.

### Tissue microarray and specimen characteristics

As part of the translational component of study #10-001857 approved by the UCLA IRB, a tissue microarray was created from 141 surgical specimens (NF-1 associated MPNST n=20, spontaneous MPNST n=33, neurofibroma n=57, schwannoma n=11, and normal nerve n=20) over a period of 27 years (1982-2009) from a single institution. Of the 53 MPNST samples, 38 were primary tumors, 10 were recurrent tumors, and 5 were metastatic (Table 1). Tissue was formalin-fixed and paraffin-embedded, and cores were selected by a sarcoma-specific pathologist (S.M.D.) and compiled in the tissue microarray. If possible, multiple cores were selected from a single tumor to account for tumor heterogeneity.

### Immunohistochemistry

Given that PD-L1 is inducible and may reflect homeostatic responses to immune activation, we contextualized our evaluation of PD-L1 with the synchronous presence CD8+ TILs in all neurogenic tissues. Cores were stained in triplicate for PDL-1, PD-1, and CD8+ TIL. Sections were deparaffinized in xylene and rehydrated in graded alcohol. Endogenous peroxidase activity was blocked with 3% hydrogen peroxide (H_2_O_2_). Antigen retrieval was performed by boiling the sections in 0.01M citric acid buffer (pH 6.0) for 15 min. Sections were first blocked with anti CD8 antibody (DAKO, M7103) at a 1:50 dilution for one hour, then incubated with Dako EnVision+ System –HRP Labelled Polymer Anti-Mouse (Dako, K4001) at room temperature for 30 minutes, incubated with DAB (3, 3′-Diaminobenzidine) for visualization, and then counterstained with Gill's hematoxylin, dehydrated in ethanol, and mounted with media. For PD-L1 and PD-1, sections were incubated in 5% normal donkey serum, and then incubated overnight at 4°C with primary antibody against PD-L1 (Spring Bioscience, Cat: M4420, 1:200) and PD-1 (Cell Marque, Cat:315M-96, 1:100). Sections were then incubated with Dako EnVision+ System –HRP Labelled Polymer Anti-Rabbit (Dako, K4003) or Anti-Mouse (Dako, K3001) at room temperature for 30 minutes, incubated with DAB (3, 3′-Diaminobenzidine) for visualization, and then counterstained with Gill's hematoxylin, dehydrated in ethanol, and mounted with media. Sections were counterstained in Gill's hematoxylin. Negative and positive control slides were included. Digital images of sections were obtained using a ScanScope XT System (Aperio Technologies Inc, Vista, CA) at 400X magnification courtesy of the UCLA Translational Pathology Core Laboratory. Protein expression scores were defined as the percentage of positively stained tumor cells relative to total tumor cells within the section, and were reviewed by a sarcoma-specific pathologist (S.M.D.). Density (number of cells stained/HPF) and intensity (grade of pigment saturation) were quantified on a scale of 0 to 3. Two criteria for staining “positivity” were utilized in parallel: “5% criteria” defined a specimen as positive if staining intensity was graded 2 or 3 with greater than or equal to 5% positive cells in any of the cores; “1% criteria” defined a specimen as positive if staining intensity was graded 2 or 3 with greater than or equal to 1% positive cells in any of the cores. Assays and staining quantification were performed blinded to the study endpoints.

### Study design

Case selection was determined prospectively, and was diversified to include malignant and benign peripheral nerve sheath tumors. The primary survival endpoint was disease specific survival of MPNST patients, defined as time from surgery to death due to disease, and the secondary survival endpoint was disease free survival, defined as time from surgery to time of tumor recurrence.

### Statistical analysis methods

Multiple cores from a single tumor were analyzed with staining scores 0-3. Low tumor scores were designated as <1, and high staining scores ≥1. Biomarker staining was compared using a Pearson Chi squared analysis (alpha ≤ 0.05) and survival analysis was performed using a Cox proportional hazards model with testing of the proportional hazards assumption. In the survival analysis, patients lost to follow up were censored at their last UCLA follow-up date. Only patients with primary MPNST who underwent R0 resection were included in the survival analysis, as survival significantly decreases with microscopic/macroscopically positive margins in patients with soft tissue sarcoma [28]. Analysis of the datasets was performed using STATA 12.0 (StataCorp., 2011).
